# Supplementary material for: Anomalous enhancement of thermoelectric power factor in multiple two-dimensional electron gas system
Source: Nat Commun. 2024 Jan 16;15:322. doi: 10.1038/s41467-023-44165-3 (PMC10791671; doi:10.1038/s41467-023-44165-3)
Supplement: Supplementary file 1 — Supplementary Information [file 41467_2023_44165_MOESM1_ESM.pdf]

Supplementary information

Anomalous enhancement of thermoelectric power factor in multiple two-dimensional electron gas system

Yuto Uematsu[1], Takafumi Ishibe[1], Takaaki Mano[2], Akihiro Ohtake[2], Hideki T. Miyazaki[2], Takeshi Kasaya[2], and Yoshiaki Nakamura[1]*

[1]*Osaka University, 1-3 Machikaneyama-cho, Toyonaka, Osaka 560-8531, Japan*

[2]*National Institute for Materials Science, 1-2-1 Sengen, Tsukuba, Ibaraki 305-0047, Japan*

*E-mail: nakamura.yoshiaki.es@osaka-u.ac.jp

Supplementary Note 1

Step-like density-of-states effect in two-dimensional electron gas system.

We describe the physical mechanism of Seebeck coefficient (S) enhancement in the step-like density-of-states (DOS) caused by confining electrons spatially along one-dimensional direction, which was proposed by Hicks and Dresselhaus[1]. We call it the step-like DOS effect. Here, we focus on two-dimensional electron gas (2DEG) system with step-like DOS. Let us compare S values of 2DEG in GaAs (2DEG-GaAs) with those of three-dimensional GaAs bulk (3D-GaAs) (Supplementary Fig. 1a). On the basis of Boltzmann transport theory, S is described under single parabolic band and relaxation time approximations as follows[2];

$$S=-\frac{k_{\mathrm{B}}}{e}\frac{\int_0^{\infty}\zeta(\zeta-\zeta_{\mathrm{F}})\frac{\partial f_0}{\partial\zeta}\tau(\zeta)D_{\mathrm{x}}(\zeta)\mathrm{d}\zeta}{\int_0^{\infty}\zeta\frac{\partial f_0}{\partial\zeta}\tau(\zeta)D_{\mathrm{x}}(\zeta)\mathrm{d}\zeta} \quad (1),$$

where ζ_{F} is reduced Fermi energy, f_0 is Fermi-Dirac distribution function, $D_{\mathrm{x}}(\zeta)$ is DOS, and the index x is "2D" or "3D" representing 2DEG-GaAs or 3D-GaAs, respectively. $D_{\mathrm{2D}}(\zeta)$ and $D_{\mathrm{3D}}(\zeta)$ are described as $m/\pi\hbar^2$ and $A\zeta^{1/2}$, respectively, where m is an effective mass of a carrier, $\hbar$ is Dirac constant, ζ is a reduced carrier energy, and A is the constant value.

Let's consider conventional 2DEG without multiplied two-dimensional electron gas effect (M2DE) in rectangular quantum well (RQW) to see the step-like DOS effect in 2D system. Supplementary Figure 1b shows S as a function of ζ_{F} in 2DEG-GaAs and 3D-GaAs. 3D-GaAs exhibited higher S than 2DEG-GaAs at the same ζ_{F}. Carrier concentration n values in 2DEG-GaAs and 3D-GaAs are described as follows;

$$n=\frac{1}{b}\int_0^{\infty}D_{\mathrm{2D}}(\zeta)f_0(\zeta)\mathrm{d}\zeta \quad \text{for 2DEG-GaAs} \quad (2),$$

$$n=\int_0^{\infty}D_{\mathrm{3D}}(\zeta)f_0(\zeta)\mathrm{d}\zeta \quad \text{for 3D-GaAs} \quad (3),$$

where b is a confinement width in 2DEG-GaAs. Now, DOS in RQW without M2DE is used as D_{2D}. The calculations were performed with b of 5 and 8 nm, which are smaller than thermal de Broglie wavelength (λ_{D}) of 16.6 nm. Supplementary Figure 1c shows n as a function of ζ_{F}. 2DEG-GaAs with b of 5 and 8 nm exhibited higher n than 3D-GaAs at the same ζ_{F}. This is because 2DEG-GaAs has larger DOS at ζ=0 than 3D-GaAs (Supplementary Fig. 1a). From ζ_{F}-S and ζ_{F}-n plots, n dependences of S were obtained (Supplementary Fig. 1d). 2DEG-GaAs with b of 5 and 8 nm exhibited higher S than 3D-GaAs at the same n. Thus, it is concluded that S enhancement by the step-like DOS effect comes from higher n of 2DEG-GaAs than that of 3D-GaAs at the same ζ_{F}. This fact is the nature of the quantum confinement effect; S enhancement comes not from the sharp onset of $D_{\mathrm{2D}}(\zeta)$ at ζ_{F}, but from the large DOS at ζ=0.

To experimentally confirm the aforementioned effect, we formed RQW samples with various well widths t_{well}, as representative 2DEG-GaAs. As shown in Supplementary Fig. 1e,

calculated and measured S both increased monotonically with t_{well} decrease, where n values of the formed RQW samples are in the range from 4.5 to 18×10^{17} cm^{-3}. The experimental data were between the calculated curves computed at n=4.5 and 18×10^{17} cm^{-3}, indicating that the experimental S tendency quantitatively agreed with the theoretically calculated one. The experimental S values of 2DEG-GaAs (S_{2D}) relative to the theoretical values of 3D-GaAs (S_{3D}) also increased with t_{well} decrease (Supplementary Fig. 1f). These results demonstrated S enhancement by the step-like DOS effect experimentally and theoretically.

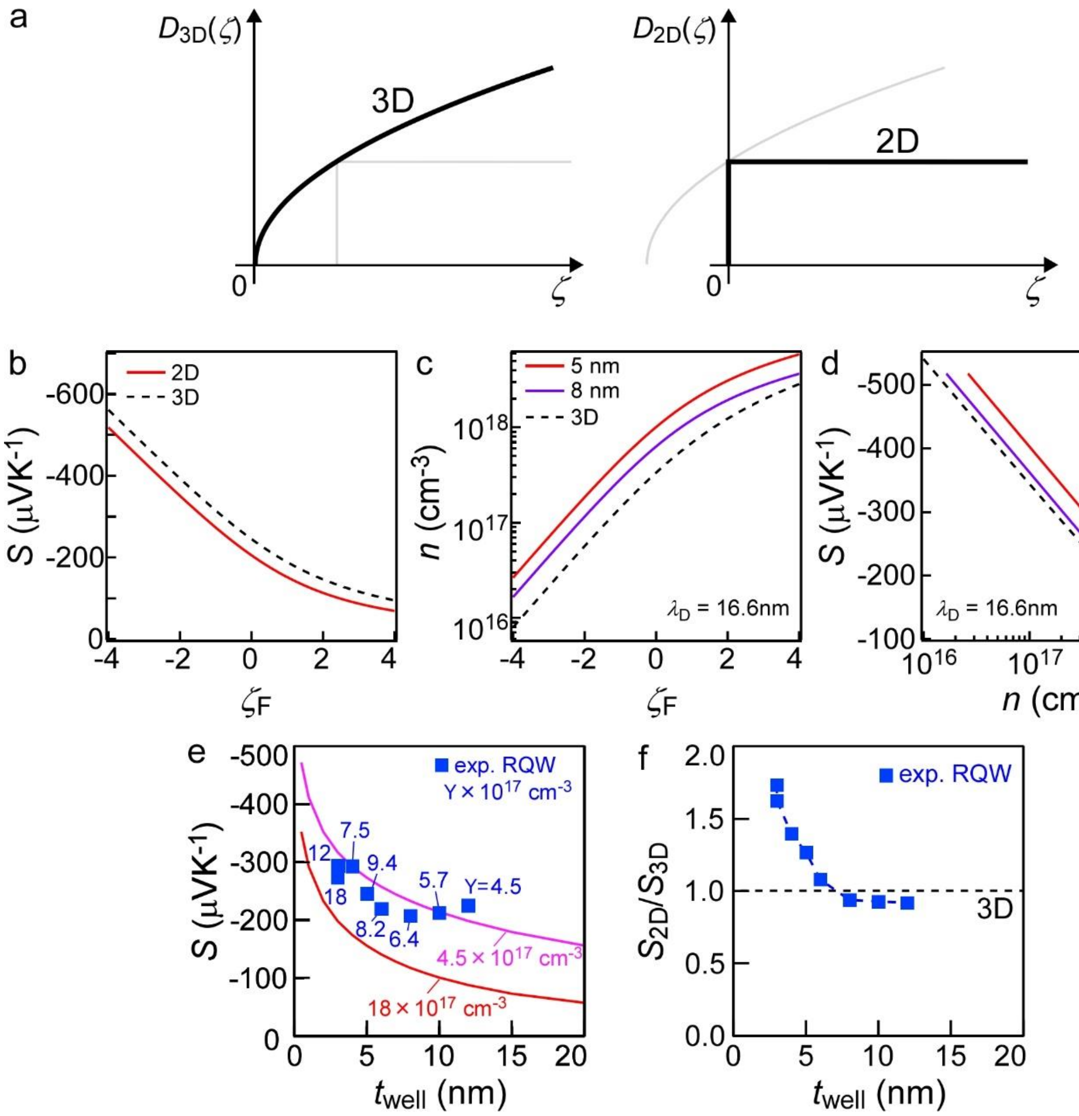

Supplementary Figure 1. The step-like density-of-states (DOS) effect in 2DEG-GaAs. **a** Schematics of DOS in 3D and 2D systems. **b, c** Reduced Fermi energy ζ_F dependences of Seebeck coefficient S (**b**) and carrier concentration n (**c**) in 2DEG-GaAs and 3D-GaAs. **d** n dependence of S in 2DEG-GaAs and 3D-GaAs. **e, f** S of 2DEG-GaAs (**e**) and S ratio between 2DEG-GaAs and 3D-GaAs (S_{2D}/S_{3D}) (**f**) as a function of well width t_{well}. In **b**-**d**, dotted and solid lines are calculated curves of 3D-GaAs and 2DEG-GaAs in rectangular quantum well (RQW) without multiplied 2DEG effect (M2DE). In **c**, **d**, thermal de Broglie wavelength λ_D of GaAs and t_{well} of 5 nm and 8 nm are described. In **e**, the two lines are calculated curves of 2DEG without M2DE (RQW) with n of 4.5×10^{17} cm^{-3} and 18×10^{17} cm^{-3}. The solid squares are experimental data of 2DEG without M2DE (RQW). In **f**, experimental data (the solid squares) are corresponding to those in (**e**).

Supplementary Note 2

Energy band diagrams of rectangular quantum well: single two-dimensional electron gas system and the clarification of single and multiple two-dimensional electron gas systems.

E_c position is changed by electrical charge based on Schrödinger Poisson equation, resulting in energy band diagram schematics of RQW and TQW[3] shown in Fig. 2a-2f in main text, respectively. In the case of RQW, the conduction band offset ΔE_c at the AlGaAs/GaAs interfaces works as an energy barrier, resulting in 2DEG formation in undoped GaAs[3].

RQW samples were formed as reference ones for single-2DEG (S-2DEG). Structure of RQW samples is shown in Supplementary Fig. 2a. We calculated energy band diagrams of RQW samples with various t_{well}. The Supplementary Figure 2b-2h show E_c and E_i as a function of z (energy band diagrams) in RQW with t_{well} of 3-12 nm, where E is a carrier energy, E_c is the energy at the conduction band bottom, E_i is the bottom energy of i-th subband, E_F is Fermi energy, and z is the distance from the interface of undoped GaAs/AlGaAs spacer along the direction perpendicular to the sample surface. The RQW samples with t_{well}=3-6 nm had only one subband, while two subbands located in the samples with t_{well}=8-12 nm. This indicates that no M2DE appeared in RQW samples with t_{well}=3-6 nm. RQW samples with t_{well}=8-12 nm had little M2DE. Actually, the contribution of M2DE in the RQW samples with t_{well}=8-12 nm is negligible because the number of subbands that can exist except the lowest one is only one.

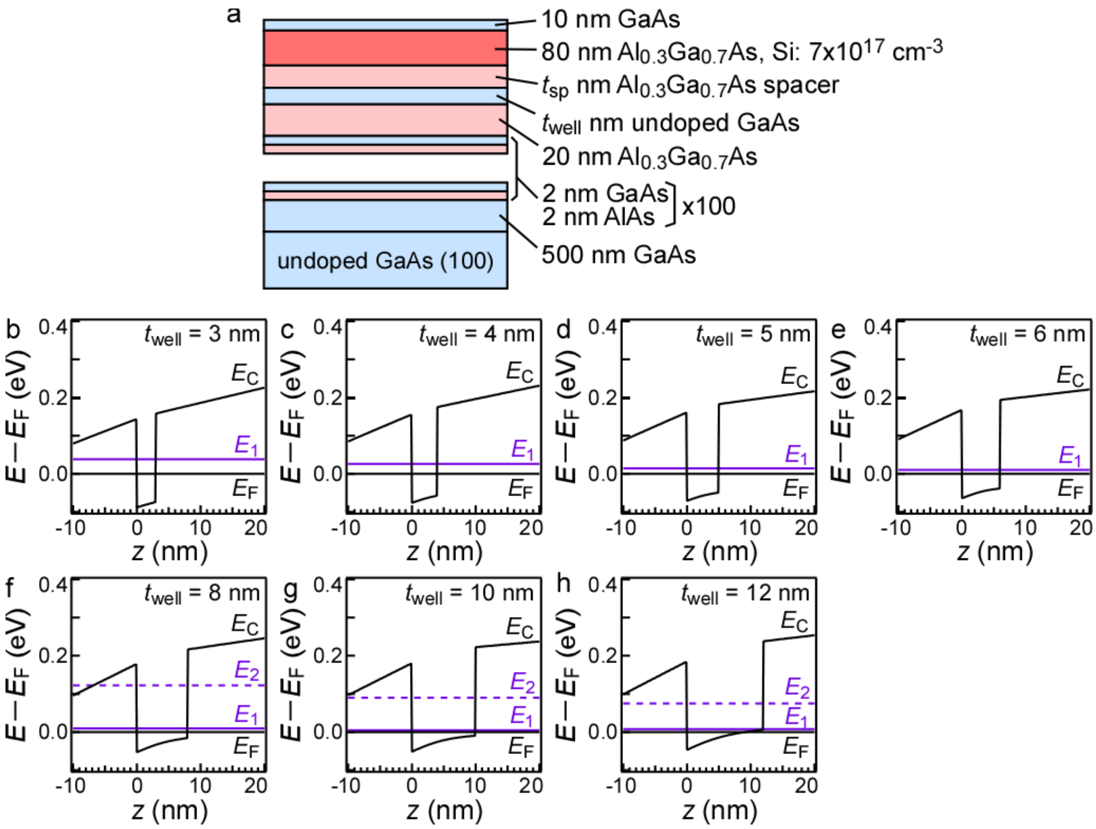

Supplementary Figure 2. Structure and energy band diagrams of rectangular quantum well (RQW) samples with various well widths t_{well}. **a** Structure of RQW samples. **b-h** Calculated conduction band bottom of 3D GaAs (E_c), the bottom energy of i-th subband (E_i), and Fermi energy E_F as a function of z (energy band diagrams) in RQW with t_{well}=3, 4, 5, 6, 8, 10, and 12 nm.

Let's discuss the origin of this small number of subbands in RQW. In the case of the infinite rectangular well, E_i is described as $\hbar^2(i\pi/t_{well})^2/(2m)$, as shown in Supplementary Figure 3a, where the energy difference between subbands (E_{i+1}-E_i) becomes larger with increasing i value. This tendency is also seen in the finite rectangular well (RQW samples). Therein, E_i easily becomes larger than ΔE_c (energy barrier height) when i value is slightly increased, as shown in Supplementary Figure 3a. Therefore, as mentioned above, there is only one subband in RQW with t_{well}=3-6 nm, and there are two subbands in RQW with t_{well}=8-12 nm. Even when two subbands exist, there is a large energy difference between E_2 and E_1. As a result, the carrier occupation ratio R_O (=n_i/n_t) of second subband is negligible in RQW, where n_i is sheet carrier concentration at the i-th subband with the bottom energy E_i and n_t is the sum of n_i, (Supplementary Fig. 3b). Therefore, "single-2DEG" refers to 2DEG system with one or two (almost one) subbands as shown by purple marks in Supplementary Fig. 3b.

On the other hand, in the case of the triangular quantum well (TQW), E_i in ideal triangular well potential is described as follows: E_i = $(3\pi(i\text{-}1/4)/2)^{2/3}\times((eF\hbar)^2/(2m))^{(1/3)}$. In contrast to the case of RQW, it represents that (E_{i+1}-E_i) becomes smaller with increasing i value in TQW. Then, this leads to large number of subbands with smaller E_i than energy barrier of TQW as shown in Supplementary Fig. 3a. Actually, 1D Poisson calculation shows 17 or more subbands in TQW and that these subbands have some values in R_O due to the small energy difference between subbands with higher i, as shown by red marks in Supplementary Fig. 3b. It should be noted that i value dependence of E_i is main origin of the number difference of subbands that can exist in TQW and RQW.

Let's summarize the difference of subbands between RQW and TQW. In TQW samples, there are a lot of subbands near E_F: e.g. at least more than 17 subbands for the TQW samples with the channel width (t_{ch}) of 8-18 nm. This indicates that TQW samples are M-2DEG system that is a 2DEG system with many subbands having some values in R_O. On the other hand, in RQW, there are only one or two subbands. Even though the two subbands exist, the energy bottom position of the second subband (E_2) is quite higher than that of the lowest subband (E_1) with reference to E_F close to E_c. Therefore, unlike TQW, carriers are difficult to be excited thermally to the higher energy subbands in RQW. As a result, RQW samples are S-2DEG system that is a 2DEG system with one or two (almost one) subbands. Therefore, comparing the thermoelectric (TE) properties of TQW with those of RQW is reasonable for the demonstration of the TE performance enhanced by M2DE in M-2DEG system.

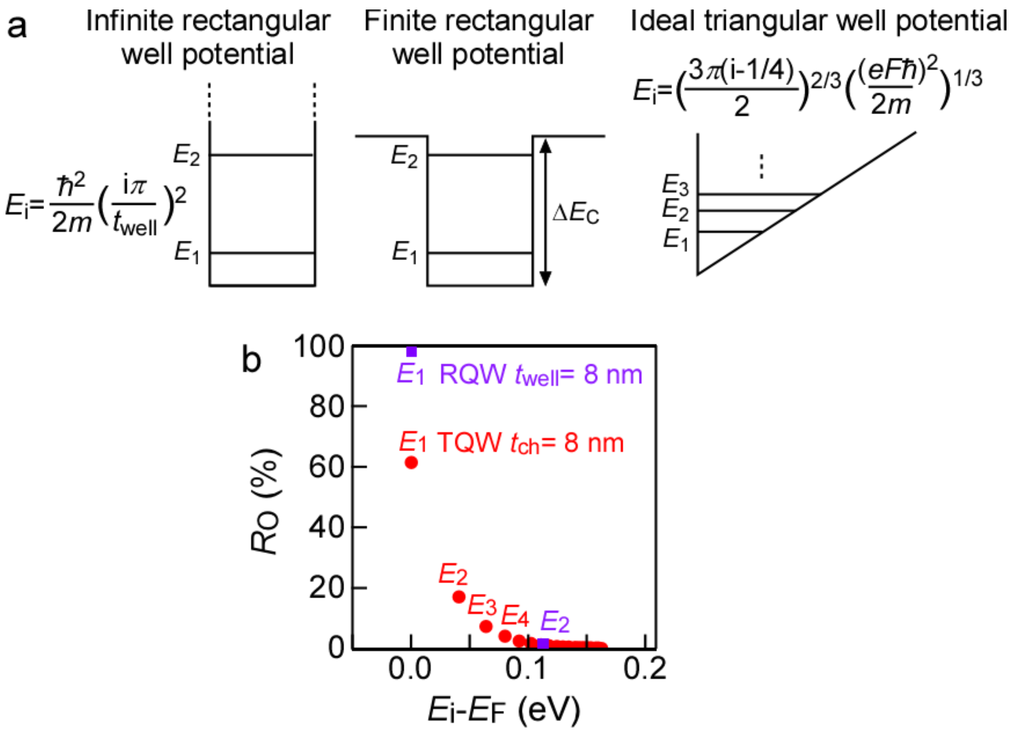

Supplementary Figure 3. Difference of the subbands between rectangular quantum well (RQW) and triangular quantum well (TQW). **a** Subbands existing in RQW and TQW. **b** Carrier occupation ratio R_O as a function of E_i-E_F (E_i: the bottom energy of i-th subband, E_F: Fermi energy) in the RQW sample with the well width t_{well}=8 nm and the TQW sample with the channel width t_{ch}=8 nm.

Supplementary Note 3

Calculated thermoelectric properties as a function of the number of subbands contributing to the electrical conduction in triangular quantum well: multiple two-dimensional electron gas.

Structure of TQW samples is shown in Supplementary Fig. 4a. The existence of a lot of subbands in TQW increases calculation cost. To reduce calculation cost while keeping calculation accuracy of TE properties, we have to remove the subbands that do not contribute electrical conduction from the calculation. Therefore, we investigated the dependence of TE properties on the number of subbands to be used for calculation. Namely, mobility (μ) and S were calculated by including the contribution of the i-th subbands from i=1 to i_m.

Supplementary Figure 4b and 4c show μ and S as a function of i_m in each TQW sample (thicknesses of spacer layers t_{sp} = 0, 2, 30, 60, and 90 nm), respectively. As for TQW samples with t_{sp}=30-90 nm, when increasing i_m, μ decreased and S increased monotonically until i_m=20. This indicates that S enhancement was brought by M2DE, while μ decreases with increase of i_m, which was caused by strong intervalley scattering between the subbands. On the other hand, both μ and S were saturated in the range of i_m>20 because of less contribution of subbands with i>20. Therefore, in this study, μ and S calculations for TQW samples with t_{sp}=30-90 nm were performed with i_m=20 which is large enough for calculation accuracy.

As for TQW samples with t_{sp} = 0 and 2 nm (small t_{ch}=8 nm), the numbers of subbands with lower E than energy barrier height were small of 17 and 19, respectively, due to the stronger confinement in small t_{ch} well. Namely, μ and S calculations for TQW samples with small t_{ch} (t_{sp} = 0 and 2 nm) were performed with i_m=17 and 19, respectively.

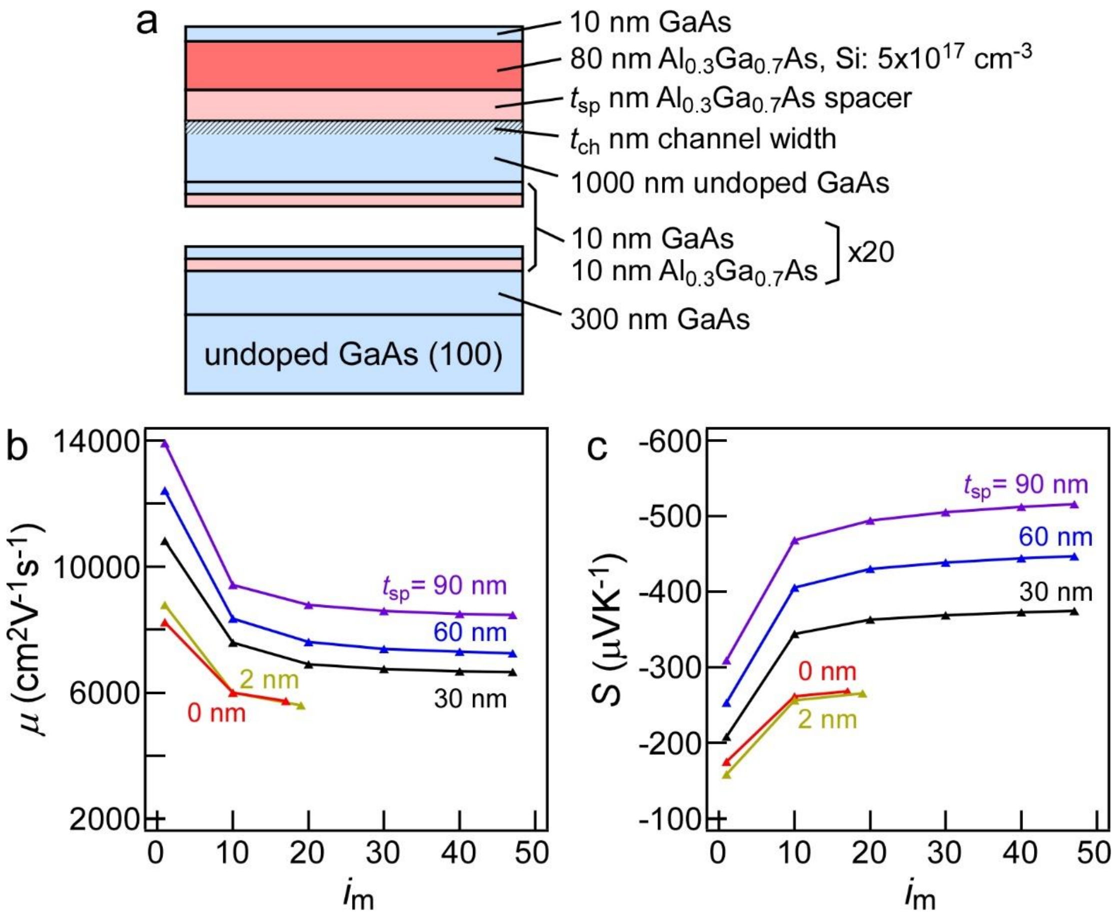

Supplementary Figure 4. Structure of triangular quantum well (TQW) samples and the number of subbands i_m dependences of the mobility μ and Seebeck coefficient S calculations in TQW. **a** Structure of TQW samples. **b, c** Calculated μ and S as a function of i_m in TQW samples the thicknesses of spacer layers t_{sp}=0, 2, 30, 60, and 90 nm.

Supplementary Note 4

Definition of the channel width in triangular quantum well.

We defined the t_{ch} in TQW as the FWHM of the calculated carrier concentration (n_{cal}) distribution vs z after obtaining energy band diagrams of TQW. This definition method has been used in the previous study[4]. Supplementary Figure 5a-5e show n_{cal} as a function of z in TQW with t_{sp} =0, 2, 30, 60, and 90 nm, respectively.

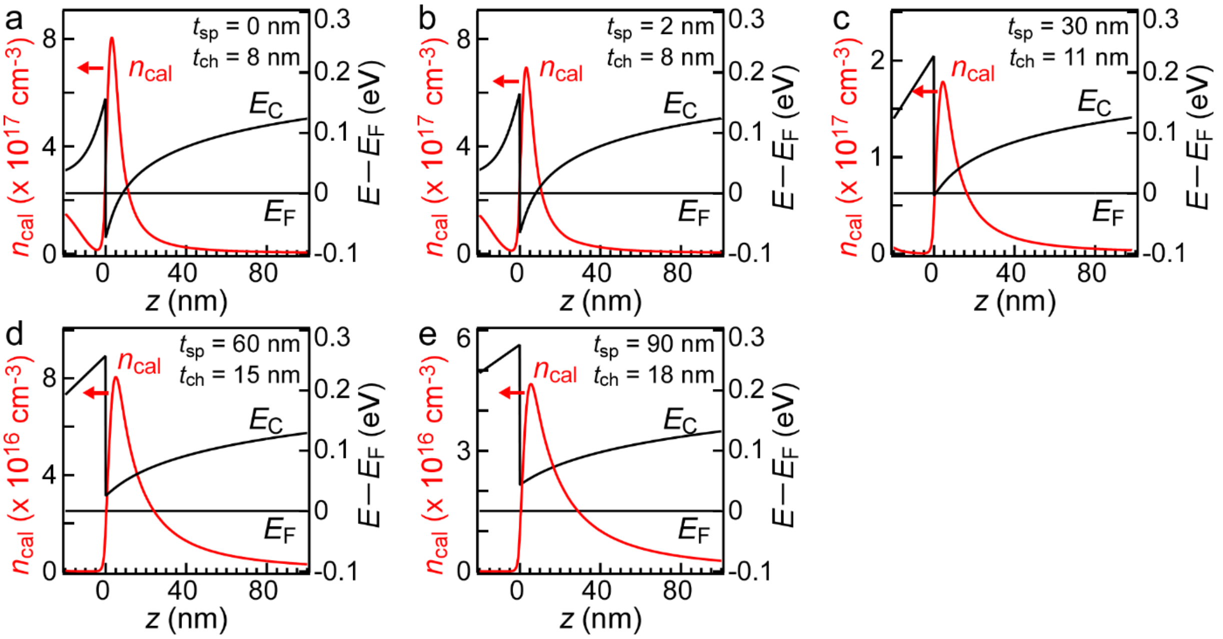

Supplementary Figure 5. Energy band diagrams of triangular quantum well (TQW). a-e Calculated carrier distribution n_{cal} and the conduction band bottom of 3D GaAs (E_c) as a function of z in the TQW with the thicknesses of spacer layers t_{sp} =0, 2, 30, 60, and 90 nm, respectively.

Supplementary Note 5

Calculation of confinement width dependence of the Seebeck coefficient at the same carrier concentration.

To discuss the quantum well shape dependence of the S enhancement effect, it is needed to see the dependence of the S enhancement on the confinement width at a certain n. So, we calculated the S of TQW and RQW at a typical n of 1×10^{18} cm^{-3} (Supplementary Fig. 6). In Supplementary Fig. 6a, t_{well} or t_{ch} are used as confinement width. In some sense, however, t_{well} and t_{ch} were defined differently from well structure and carrier distribution broadening, respectively. Then, in Supplementary Fig. 6b, the same definition for confinement width in TQW and RQW are used; namely, carrier distribution broadenings in TQW and RQW (t_{br} = full width at half maximum of n_{cal}) are used as confinement width. Supplementary Fig. 6a, 6b both show that S of TQW is larger than that of RQW over a wide range of the confinement width owing to M2DE in TQW, while in very small width, the S value of RQW is almost the same as that of TQW due to the strong confinement in RQW. Carrier mobility of TQW with single-sided interface is larger than that of RQW with double-sided interfaces because of the less carrier scattering at single-sided interface (Fig. 3b in main text). As a result, high S and high μ in TQW are expected by M2DE and modulation doping effect, bringing the larger enhancement in power factor.

There is the aforementioned discrepancy in the confinement width dependences of the S of TQW and RQW. This comes from the mechanism difference between M2DE and step-like DOS effect. Mechanism of the step-like DOS effect is reported by Hicks and Dresselhaus[1], which leads to the strong confinement width dependence. On the other hand, M2DE is caused by the relatively large participation rate of higher-energy carriers in the carrier conduction. This depends on the subband number and E_i, leading to the relatively low confinement width dependence.

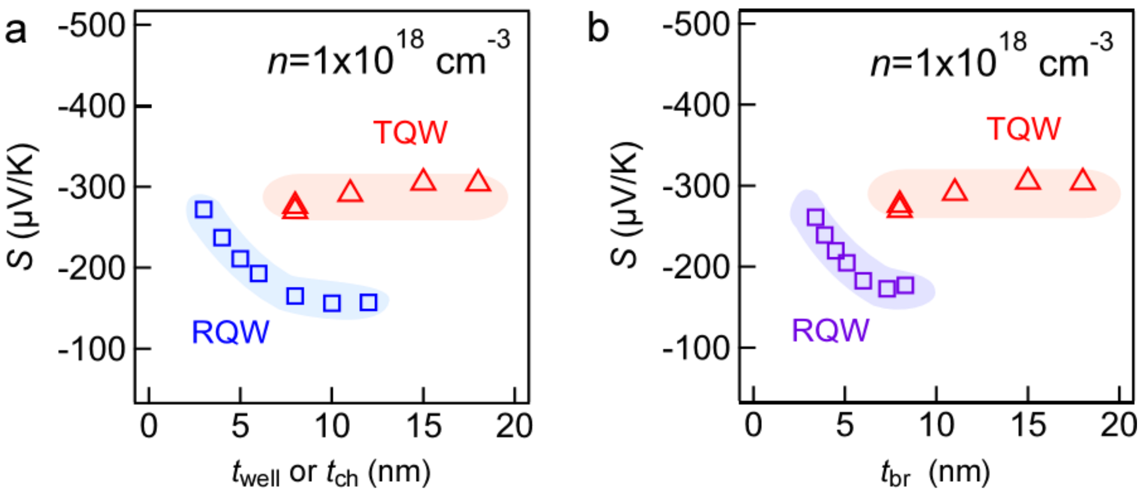

Supplementary Figure 6. Calculation of confinement width dependence of Seebeck coefficient *S* at the same carrier concentration *n*. **a, b** *S* is calculated as a function of the confinement width (t_{well} for rectangular quantum well (RQW) and t_{ch} for triangular quantum well (TQW) in (a), and t_{br} for RQW and TQW in (b)). t_{br} is a full width at half maximum of calculated carrier distribution n_{cal}. The open triangles and squares are calculated results in the case of TQW and RQW, respectively, at a typical *n* of 1×10^{18} cm^{-3}. This calculation is for TQW under the virtual sample condition because experimentally, *n* of 10^{18} cm^{-3} and t_{ch} of 11-18 nm cannot be realized at the same time technically in the case of GaAs (in terms of the triangle potential shape).

Supplementary Note 6

Temperature dependence of the Seebeck coefficient of triangular quantum well.

To demonstrate M2DE, we calculated and measured the temperature dependence of the S of TQW. Supplementary Figure 7a shows the calculated T-S curve of TQW, which shows a gradual change even around the temperature corresponding to the energy difference E_2-E_1 (=$k_B T$). It might be expected that temperature dependence of Seebeck coefficient shows a sharp change at the temperature corresponding to the energy difference E_2-E_1, but according to the calculation, it is actually changed gradually. The reason is mentioned as follows; the subband bottom is discrete, but the subband itself has a continuous energy due to the energy at the x and y directions. Therein, carriers distribute in this step-like but continuous density of states in accordance with the Fermi-Dirac function. And then, change of Seebeck coefficient coming from step-like DOS change at E_2 is too small to detect (Supplementary Fig. 7a). We also calculated R_O. Therein, R_O of the first subband at 160 K is larger than that of 300 K (Supplementary Fig. 7b). However, some carriers exist in higher subbands even at low temperature because n_i gradually reduces based on the temperature dependence of Fermi-Dirac distribution. Therefore, M2DE is gradually suppressed at low temperature. Supplementary Figure 7c shows the experimental results of temperature dependence of the Seebeck coefficient (the solid triangles) along with the calculated results (the open squares). This demonstrates that the calculation and experimental results agreed well. This proved the existence of M2DE in terms of temperature dependence.

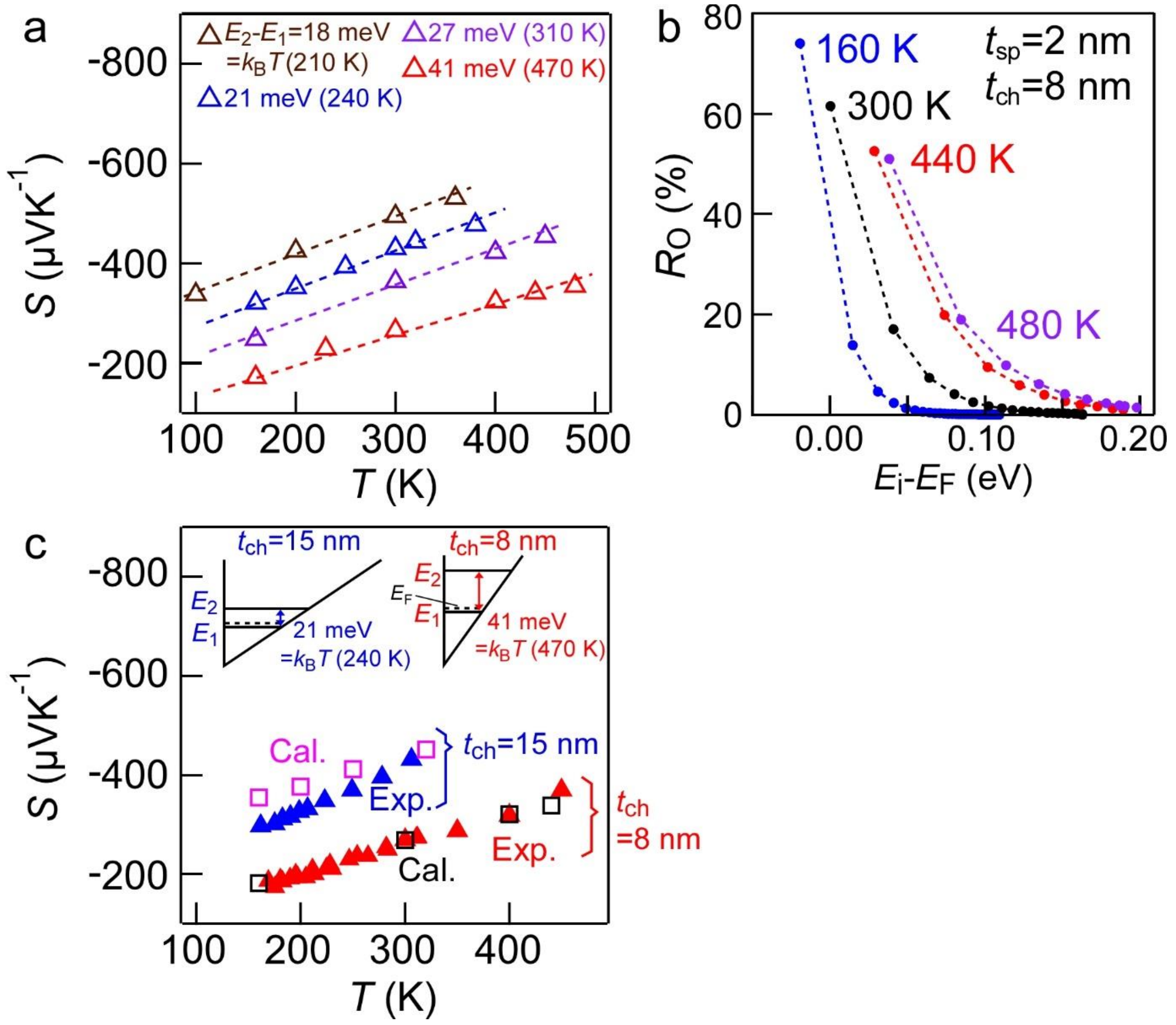

Supplementary Figure 7. Temperature *T* dependences of Seebeck coefficient *S* and carrier occupation ratio R_O. **a** Calculated *T*-*S* data of triangular quantum well (TQW) with various channel widths t_{ch} (various E_2-E_1 = 18, 21, 27, 41 meV) (E_i: the bottom energy of *i*-th subband). They are simple calculations with constant carrier concentration *n* measured at 300 K. **b** R_O as a function of E_i-E_F in the TQW with t_{ch}=8 nm at various temperatures (160, 300, 440, 480 K). **c** Experimental *T*-*S* data with the calculated ones of TQW with t_{ch} of 8 and 15 nm (E_2-E_1 = 41 and 21 meV, respectively). Calculated data were computed using *n* measured at each temperature. The solid triangles are experimental data, and the open squares are calculated ones.

Supplementary Note 7

Removing the contribution of the doped AlGaAs layer to electrical conduction in triangular quantum well and rectangular quantum well samples.

In TQW and RQW samples, modulation doping was performed by inserting Si-doped AlGaAs layer. Therefore, this doped layer could contribute to the electrical conduction. Here, we remove the contribution of the doped layer using a parallel conduction model as follows:

$$n_{s\_all}=\frac{(\sigma_{s\_2DEG}+\sigma_{s\_DL})^2}{\sigma^2_{s\_2DEG}/n_{s\_2DEG}+\sigma^2_{s\_DL}/n_{s\_DL}} \quad (4),$$

$$S_{2DEG}=\frac{\sigma_{s\_all}S_{all}-\sigma_{s\_DL}S_{DL}}{\sigma_{s\_2DEG}} \quad (5),$$

$$\sigma_{s\_all}=\sigma_{s\_2DEG}+\sigma_{s\_DL} \quad (6),$$

where n_{s_α}, S_{α}, and σ_{s_α} are sheet carrier concentration, Seebeck coefficient, and sheet electrical conductivity, respectively. The index α is "all", "DL", or "2DEG" representing the measured value of the TQW and RQW samples, the measured value of the doped layer sample, and the estimated value of 2DEG channel in TQW and RQW samples, respectively. Therein, the measured values of the doped layer sample were obtained by characterizing the Si-doped $Al_{0.3}Ga_{0.7}As$ sample without TQW and RQW. For example, in the TQW sample with t_{sp}=2 nm, n_{s_all}, S_{all}, and σ_{s_all} were measured to be 1.1×10^{12} cm^{-2}, -279 μVK^{-1}, and 1.1×10^{-3} $\Omega^{-1}sq.$, respectively. In addition, n_{s_DL}, S_{DL}, and σ_{s_DL} of the Si-doped $Al_{0.3}Ga_{0.7}As$ layer (80 nm) sample were measured to be 2.6×10^{11} cm^{-2}, -407 μVK^{-1}, and 3.4×10^{-5} $\Omega^{-1}sq.$, respectively. Using Eq. 4-6, n_{s_2DEG}, S_{2DEG}, and σ_{s_2DEG} were estimated to be 1.0×10^{12} cm^{-2}, -275 μVK^{-1}, and 1.1×10^{-3} $\Omega^{-1}sq.$, respectively. In the same manner, the contributions of the doped layers in all TQW and RQW samples were removed using parallel conduction model. Supplementary Figure 8 shows S and electrical conductivity σ as a function of measured Hall carrier concentration n. There were almost no value differences between before (the open marks) and after removing the contributions of the doped layers (the solid marks). Namely, it was demonstrated that the contributions of the doped layers to the TE properties were negligibly small. In the main text, we used the experimental data without the contribution of the doped layers.

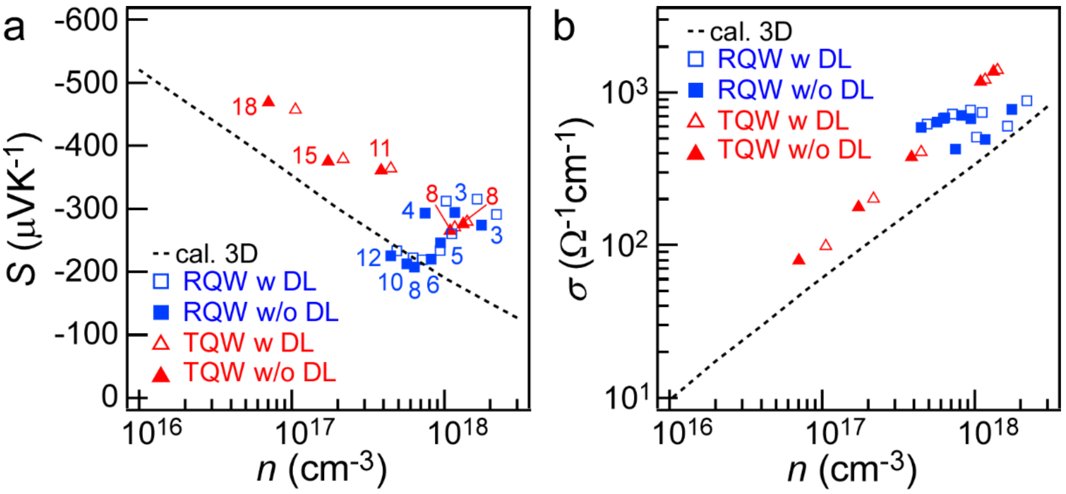

Supplementary Figure 8. Experimental data with and without the contribution of the doped layers in triangular quantum well (TQW) and rectangular quantum well (RQW) samples. **a, b** Seebeck coefficient S and electrical conductivity σ as a function of carrier concentration n in all TQW and RQW samples, respectively. The open (solid) triangles and squares are experimental data with (without) the contributions of the doped layers in TQW and RQW samples, respectively. The samples with and without the contributions of the doped layers are named as H w DL and H w/o DL, respectively, where H is the sample name. The broken lines represent the calculation curve of 3D GaAs. In **a**, the channel widths t_{ch} of TQW or well widths t_{well} of RQW are represented around the experimental data points.

Supplementary References

1. Hicks, L. D. & Dresselhaus, M. S. Effect of quantum-well structures on the thermoelectric figure of merit. *Phys. Rev. B* **47**, 12727-12731 (1993).
2. Chen, G. & Shakouri, A. Heat transfer in nanostructures for solid-state energy conversion. *J. Heat Transfer* **124**, 242-252 (2002).
3. Davies, J. H. *The Physics of Low-Dimensional Semiconductors: An Introduction* (Cambridge Univ. Press, Cambridge, England, 1997).
4. Ohta, H. *et al*. Giant thermoelectric Seebeck coefficient of a two-dimensional electron gas in $SrTiO_3$. *Nat. Mater*. **6**, 129-134 (2007).
